# Intrarenal Single-Cell Sequencing of Hepatitis B Virus Associated Membranous Nephropathy

**DOI:** 10.3389/fmed.2022.869284

**Published:** 2022-07-22

**Authors:** Leilin Yu, Wei Lin, Chanjuan Shen, Ting Meng, Peng Jin, Xiang Ding, Peter J. Eggenhuizen, Joshua D. Ooi, Rong Tang, Wannian Nie, Xia Li, Xiangcheng Xiao, Yong Zhong

**Affiliations:** ^1^Department of Nephrology, Xiangya Hospital, Central South University, Changsha, China; ^2^Jiujiang Traditional Medicine Hospital, Jiujiang, China; ^3^Department of Pathology, Xiangya Hospital, Central South University, Changsha, China; ^4^Key Laboratory of Biological Nanotechnology of National Health Commission, Xiangya Hospital, Central South University, Changsha, China; ^5^Department of Hematology, The Affiliated Zhuzhou Hospital Xiangya Medical College, Central South University, Changsha, China; ^6^Department of Organ Transplantation, Xiangya Hospital, Central South University, Changsha, China; ^7^Centre for Inflammatory Diseases, Monash University, Clayton, VIC, Australia

**Keywords:** hepatitis B virus (HBV), membranous nephropathy, single-cell RNA sequencing, endothelial cells, podocytes

## Abstract

To date, the pathogenesis of hepatitis B virus (HBV)-associated membranous nephropathy (MN) remains elusive. This study aimed to decipher the etiopathogenesis of HBV-associated MN by performing single-cell RNA sequencing (scRNA-seq) of kidney biopsy specimens from a patient with HBV-associated MN and two healthy individuals. We generated 4,114 intrarenal single-cell transcriptomes from the HBV-associated MN patient by scRNA-seq. Compared to healthy individuals, podocytes in the HBV-associated MN patient showed an increased expression of extracellular matrix formation-related genes, including *HSPA5, CTGF*, and *EDIL3*. Kidney endothelial cells (ECs) in the HBV-associated MN were enriched in inflammatory pathways, including NF-kappa B signaling, IL-17 signaling, TNF signaling and NOD-like receptor signaling. Gene ontology (GO) functional enrichment analysis and Gene Set Variation Analysis (GSVA) further revealed that differentially expressed genes (DEGs) of ECs from the HBV-associated MN patients were enriched in apoptotic signaling pathway, response to cytokine and leukocyte cell-cell adhesion. The up-regulated DEGs in glomerular ECs of HBV-associated MN patients were involved in biological processes such as viral gene expression, and protein targeting to endoplasmic reticulum. We further verified that the overexpressed genes in ECs from HBV-associated MN were mainly enriched in regulation of protein targeting to endoplasmic reticulum, exocytosis, viral gene expression, IL-6 and IL-1 secretion when compared with anti-phospholipase A2 receptor (PLA2R)-positive idiopathic membranous nephropathy (IMN). The receptor-ligand crosstalk analysis revealed potential interactions between endothelial cells and other cells in HBV-associated-MN. These results offer new insight into the pathogenesis of HBV-associated MN and may identify new therapeutic targets for HBV-associated MN.

## Introduction

Hepatitis B virus (HBV)-associated glomerulonephritis (HBV-GN) is highly prevalent in most Asian endemic areas. Although whether there is a direct causal relationship between HBV and the occurrence of kidney diseases remains unsettled, HBV infection has been linked to the development of various kidney diseases. Membranous nephropathy (MN) is the most common subtype among them. MN frequently leads to proteinuria and nephrotic syndrome, which can be categorized into primary form and secondary form based on its etiologies. The primary form of MN is caused by autoimmune disease with relatively slow progression. As reported by Moroni and Ponticelli (1), this form accounts for up to ~70% of MN cases. The secondary form is caused by other pathogenic factors, such as infections, drugs, toxins, and tumors (2–4). Particularly, secondary MN is frequently seen in patients with chronic HBV infections. The formation mechanisms of HBV-associated MN are complicated and involve the following four aspects. First, intracellular viral infection can cause cytopathic effects. Second, the deposition of HBV antigen and antibody immune complexes in kidney tissues may lead to renal cell injury. Third, virus-induced specific immune effector mechanisms, such as specific T lymphocytes or antibodies, could also cause kidney damage. Lastly, virus-induced cytokine production may have indirect effects on renal tissue (5). MN is the most common renal lesion associated with HBV infection, and 70% of them are positive for anti-PLA2R. Several studies have put forward the hypothesis that HBV can cause anti-PLA2R that leads to MN (6). However, the pathological processes underlying HBV-associated MN remain unclear. Therefore, the relationship between HBV-associated MN and MN needs further exploration.

Single-cell RNA-sequencing (scRNA-seq) is a powerful tool that facilitates transcriptome profiling of thousands of single cells simultaneously to determine the transcriptomic variations present in various diseases. It also enables the comprehensive description of kidney diseases at cellular resolution, alongside ascertaining cell subtypes and demonstrating differences on a molecular level (7). This technique has been applied in the investigation of various kidney-related diseases, including acute or chronic kidney injury, kidney cell carcinoma, lupus nephritis, and diabetic nephropathy (8). Compared to the bulk RNA-seq that measures average gene expression of all cells in the tissues (9), scRNA-seq has many advantages as it enables simultaneous inquiry of diverse cell types, their transcriptomic profiles and signaling pathways. Therefore, it has been considered to be a useful tool that can define comprehensive gene sets at the single-cell level (10–15). Since the Humphreys group adopted scRNA-seq in healthy adult kidneys and one kidney transplant biopsy, there have been many advances in single-cell sequencing technology in mapping the human kidney transcriptome (16). Recently, we have taken advantage of this technique to generate renal atlases in IgA nephropathy (17) and membranous nephropathy (18).

In this study, we sought to apply scRNA-seq to kidney biopsy samples from a patient with HBV-MN and healthy individuals to identify transcriptome alterations at the cellular level in HBV-MN, to further determine the crucial molecular mechanisms involved in the development of this disease.

## Methods

### Ethical Approval and Consent

This study was approved by the Medical Ethics Committee of Xiangya Hospital, Central South University (approval number 201711836). The conduct of experiments adhered to pre-determined study protocols and the International Ethical Guidelines for Research Involving Human Subjects (detailed in the Declaration of Helsinki). Participants provided written consent or had the agreement of their respective guardians.

### Clinical Sample Procurement

Kidney biopsy specimens were obtained from one recently diagnosed HBV-associated MN patient in the Department of Nephrology, Xiangya Hospital at Central South University. The samples themselves were acquired *via* conducting renal core needle biopsies from nephrotic syndrome subjects in parallel with the positive serum anti-PLA2R antibody and were taken *via* the use of 18-gauge needles. The biopsy tissues were cleaned and sterilized with phosphate-buffered saline (PBS) after being extracted.

### Single-Cell Suspension Preparation

The extracted fresh kidney tissue samples were processed and dissociated into single-cell suspensions using a previously described method (18).

### scRNA-Seq Library Preparation and Sequencing

The single-cell suspension was prepared by re-suspending at a concentration of 1 × 10^5^ cells/mL in PBS, after which it was added to the microfluidic chip. Moreover, following the manufacturer's protocols, the scRNA-seq libraries were prepared by drawing upon the Singleron GEXSCOPE Single Cell RNA-seq Library Kit (Singleron Biotechnologies) which is comprised of mRNA (unique molecular identifier (UMI) and mRNA trapping, cell lysis, reverse transcription mRNA into cDNA and amplification, labeling cells barcode and fragment cDNA. Individual libraries were diluted to 4 nM and pooled for sequencing. Pools were sequenced on an Illumina HiSeq X with 150 bp paired end reads.

### Preprocessing of Raw Data

To generate gene expression profiles, raw reads of samples were processed to generate an internal pipeline, poly-A tails and adapters were trimmed (fastp V1), and read two was aligned to GRCh38 in conjunction with ensemble version 02 gene annotation (fastp 2.5.3a and featureCounts 1.6.2). Reads that possessed the same UMI, gene, and barcode were grouped to ascertain the number of UMIs per gene per cell.

### Quality Control

Quality control was performed after the expression matrix was generated after which Seurat V3.1.2 was performed. As for quality control, cells with 0–30,000 UMIs and <50% mitochondrial expression percentage were selected for downstream analysis. In addition, cells with 0–6,000 genes within samples and 200–5,000 genes between samples were also important factors in quality control.

### Dimension Reduce and Clustering Analysis

The Cele Scope software obtained from git clone https://gitee.com/singleron-rd/celescope.git latest was used to perform cell barcode counting. After obtaining a clean data expression matrix, the dimension reduction operation (Uniform Manifold Approximation and Projection, UMAP) to visualize the cell distribution status and clustering analysis were performed. This was achieved by using Seurat's (http://satijalab.org/seurat/, R package, v.3.0.1) SNN (shared nearest neighbor) model. The top 20 principal components were used for the dimension reduction. Furthermore, Seurat's Subset Data function was utilized to complete a sub-clustering analysis of endothelial cells and myeloid immune cells.

### Differentially Expressed Genes Analysis

To identify potential DEGs, we used the Seurat FindMarkers function based on the Wilcox likelihood-ratio test with their default parameters. We selected the genes expressed in over 10% of total cells in a cluster and with an average log (Fold Change) value > 0.25 and the *p* < 0.05 as DEGs. In addition, the DEGs were ascertained between the HBV-MN transcriptional profile and the donor subjects as well as between two patients with IMN that came from our previous study studies (17, 18) and the HBV-associated MN subject.

### Cell Type Annotation

The cell types are firstly automatically annotated by singleR, and then the annotation results are manually reviewed according to the cannonical marker gene. The reference database SynEcoSysTM, as well as canonical markers of literature references, defined cells of each cluster. SynEcoSysTM is a single-cell database platform which can provide rapid and accurate cell annotation services based on CellMakerDB, PanglaoDB, and recently published works of literature. The top DEGs found in the cluster by the pattern of canonical markers define cell types accordingly. Seurat (http://satijalab.org/seurat/, R package, v.3.0.1) was employed to display the expression of markers *via* Heatmaps/violin plots. Doublet cells were identified as expressing markers for different cell types and removed manually.

### Gene Ontology and Kyoto Encyclopedia of Genes and Genomes Enrichment Analysis

To investigate the potential functions of DEGs, GO and KEGG analysis were used with the “clusterProfiler” R package. Pathways with *p*_adj_ value <0.05 were considered as significantly enriched. Gene Ontology gene sets, including molecular function (MF), biological process (BP), and cellular component (CC) categories, were used as reference. The 50 hallmark gene sets derived from the MSigDB databases (https://www.gsea-msigdb.org/gsea/msigdb) were utilized for generalized subsystem vibrational analysis (GSVA) pathway enrichment analysis, and the average gene expression of each cell type was used as input data.

### Cell Interaction Analysis

Cellphone DB was performed to display ligand-receptor cell interaction between kidney cells based on known ligand-receptor pairs. Cutoffs for the mean log gene expression distribution of all genes were based on thresholding individual ligand or receptor expression per cell type. The significant cell-cell interactions were defined when the *p* < 0.05 and the average log expression > 0.1, which were visualized with the circlize (0.4.10) R package.

### Immunofluorescence Assay

Renal tissues were first treated with xylene for dewaxing the paraffin-embedded sections in water, then repairing antigen in a microwave oven by placing the tissue sections in ethylenediaminetetraacetic acid (EDTA) antigen repair buffer (pH = 8.0). After natural cooling, the sections were placed in PBS, shaken, and washed on a decolorization shaking table three times (5 min each time), followed by drawing a circle and sealing with hydrogen peroxide. Primary antibodies against AQP1 (GB11310, Wuhan Servicebio Technology, 1:5000), HSPA5 (GB11492, Wuhan Servicebio Technology, 1:50), IGFBR7 (ab8451, Abcam, 1:3000), JUN (ab8451, Abcam, 1:500), SELE (GB113279, Wuhan Servicebio Technology, 1:100), TNFAIP3 (ab8451, Abcam, 1:50) were diluted in blocking buffer and then incubated overnight. After incubation, slides were washed with 0.1% PBST three times (20 min each time), and the secondary antibodies were diluted in a 1:500 ratio and incubated for 2 h. The slides were further washed by 0.1% PBST after incubation another three times. Images were acquired by using fluorescence microscopy and captured images with a 40X water objective lens (NA 1.25).

## Results

A 64 year-old Asian man showed repeated edema and proteinuria for 3 years, and he had been suffering from hepatitis B for more than 8 years. He had a creatinine level of 75 μmol/L and an eGFR level of 92.15 mL/min/1.73 m^2^. The results of serum anti-PLA2R antibodies were 39.46RU/mL, and proteinuria was 1.8 g/24 h. To further clarify the etiology, a kidney biopsy was performed to ascertain the presence of histologic evidence that is suggestive of hepatitis B virus membranous nephropathy. The results revealed the diffused formation of subepithelial “spikes,” also known as glomerular basement membrane heterogeneous thickening *via* light microscopy (Supplementary Figure 1). Immunofluorescence microscopy observation exhibited a granular immunofluorescence deposition of the HBsAg, HBcAg, IgG, IgM, C3, and C4 in HBV-associated MN disease (Supplementary Figure 1). The resulting histologic diagnosis was hepatitis B virus membranous nephropathy and chronic viral hepatitis B. Before the kidney biopsy, the HBV-associated MN patient did not receive any medication aside from RAAS inhibitors.

### Cell Lineage Identification

**Figure 1:**
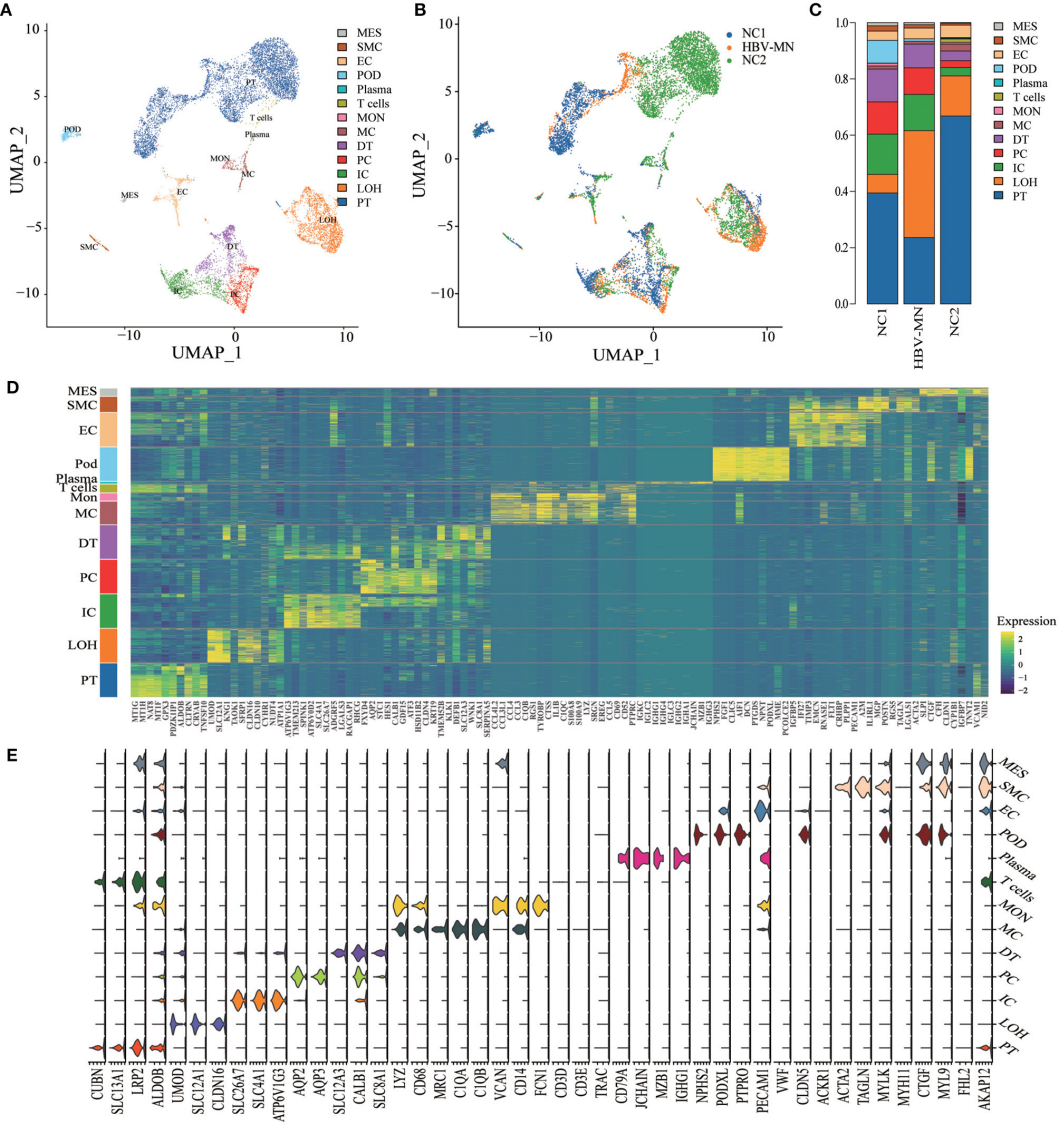
Cell lineage analysis by comprehensive single-cell RNA-sequencing in HBV-MN and healthy subjects. **(A)** Thirteen distinct cell clusters were visualized by UMAP plotting, with each cell color-coded for its associated subtypes. The color of the cells represents group origin. **(B)** UMAP plot of cell clusters from different subjects of HBV-MN and healthy subjects. The color of cells reflected the individual origin. **(C)** Bar plots of the percent contribution of cell clusters in kidneys from different subjects. Blocks represented different subjects, and block height was in proportion to the number of cells. **(D)** Heatmap of the top 10 most differentially expressed genes in each cluster to identify mutually exclusive gene sets, which were then used to determine the cell lineage of each cluster. Each column represented a cell cluster, and each row corresponded to a marker gene for the individual cluster. Transcript abundance ranges from low (purple) to high (yellow). **(E)** Violin plot of selected marker genes that identified the clusters generated by UMAP plotting. It was colored by different cell subtypes. PT, proximal tubule cells; LOH, loop of Henle cells; PC, principal cells; IC, intercalated cells; DT, distal tubule cells; EC, endothelial cells; Pod, podocytes; MES, mesangial cell; MC, macrophages; Mon, monocytes; SMC, smooth muscle cell.

In total, we sequenced 4,114 single cells in HBV-associated MN; 2,318 kidney cells in HBV-associated MN patient and 9,182 kidney cells in healthy subjects were retained for the downstream analysis (Supplementary Table 1). A total of thirteen distinct cell clusters were retained *via* the use of unsupervised clustering analysis and subsequently labeled following specific lineage marker gene expression (Figure 1A, Supplementary Table 2). The distribution of cells from different samples was exhibited by uniform manifold approximation and projection (UMAP) (Figure 1B). Bar plots represented the frequency of cell clusters in the kidney of each subject, which clearly defined the characteristics of each cell cluster. In contrast, the difference was calculated for each subject (Figure 1C). The reliability of the cell classification method was shown by the expression of the top 20 marker genes for each cell population among 13 clusters (Figure 1D, Supplementary Table 3). Known kidney cell marker gene expression patterns were subsequently analyzed to accurately define each cell cluster (Table 1). Figure 1E shows each cell population identified by selected cell lineage-specific marker gene expression.

**Table 1:** Markers for different cell types based on their cell lineage.

**Marker**	**Cell types**	**References**
CD2	T Cells	Izar et al. (19)
CD3E		Stewart et al. (20)
CD3D		Stewart et al. (20)
TRAC		Wu et al. (16)
CD79A	Plasma cells	Reyes et al. (21)
JCHAIN		Gate et al. (22)
MZB1		Zhang et al. (23)
IGHG1		Durante et al. (24)
LYZ	Monocytes	Reyes et al. (21)
VCAN		Reyes et al. (21)
CD14		Schafflick et al. (25)
FCN1		Zheng et al. (26)
LYZ	Macrophages	Habermann et al. (27)
CD68		Malone et al. (28)
MRC1		Bian et al. (29)
C1QA		Zeng et al. (30)
C1QB		Malone et al. (28)
ACTA2	Smooth muscle cells	Zhang et al. (31)
TAGLN		Merrick et al. (32)
MYLK		van Zyl et al. (33)
MYH11		Asp et al. (34)
MYL9	Mesangial cells	Panglaodb
FHL2		Wu et al. (35)
AKAP12		Panglaodb
NPHS2	Podocytes	Stewart et al. (20)
PODXL		Wu et al. (16)
*PTPRO*		Stewart et al. (20)
PECAM1	Endothelial cells	Der et al. (36)
VWF		Vijay et al. (37)
CLDN5		Motazedian et al. (38)
ACKR1		Young et al. (39)
CUBN	Proximal tubule cells	Wu et al. (16)
SLC13A1		Stewart et al. (20)
LRP2		Malone et al. (28)
ALDOB		Stewart et al. (20)
UMOD	Loop of Henle cells	Wu et al. (16)
SLC12A1		Stewart et al. (20)
CLDN16		Stewart et al. (20)
SLC12A3	Distal tubule cells	Wu et al. (16)
CALB1		Der et al. (36)
SLC8A1		Wu et al. (16)
SLC26A7	Intercalated cells	Wu et al. (16)
SLC4A1		Young et al. (39)
ATP6V1G3		Ransick et al. (40)
AQP2	Principal cells	Wilson et al. (12)

### Identification of Changes in Gene Expression in Kidney Cells of HBV-Associated-MN Patients

To unveil the gene expression changes occurring in the parenchymal cells of the kidney, differential expression analysis was performed with the transcriptomes between the HBV-associated MN patient and healthy individuals. DEGs of renal cells in the glomerulus, tubules, and immune cells were included in Supplementary Tables 4–6, respectively.

**Figure 2:**
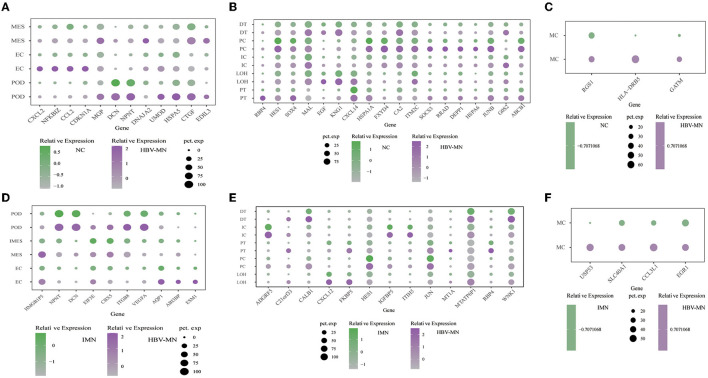
DEGs in the kidney cells between HBV-MN and healthy/IMN subjects. **(A–C)** Representative DEGs in glomerular cells, tubular cells, and immune cells comparing the HBV-MN patients to the healthy donors, respectively. pct. exp, percentage of cells expressing gene. **(D–F)** Representative DEGs in glomerular cells, tubular cells, and immune cells comparing the HBV-MN patients to IMN subjects. pct. exp, percentage of cells expressing gene, respectively.

Representative DEGs were defined in both glomerular intrinsic cells (Figure 2A), tubular intrinsic cells (Figure 2B), and immune cells (Figure 2C) via comparing the gene expression levels between HBV-associated MN and the control subjects.

Comparison of podocytes from HBV-associated MN and control subjects revealed upregulation of genes that are involved in protein processing, protein complex binding and extracellular matrix. It was reported for the first time in our study that several genes including *HSPA5, CTGF*, and *EDIL3* were overexpressed in podocytes of HBV-MN (Figure 2A). *HSPA5* was also a master regulator of the anti-apoptotic unfolded protein response signaling network (41–43). Notably, HBV-MN's podocyte highly expressed *HPSA5* (Figure 2A), which is a gene expressed in the endoplasmic reticulum and is also expressed highly in tubular cells of IMN. We verified the upregulation of *HSPA5* at the protein level in kidney biopsy tissue from different HBV-MN patients by immunofluorescence staining (Supplementary Figure 3B).

Previous studies demonstrated that *CTGF* is involved in the progression of kidney fibrosis and modulates the expression of inflammatory mediators *via* various signaling pathways (44). More importantly, several reports have shown the upregulation of CTGF expression in various instances of human glomerulonephritis, including IgA nephropathy and crescentic glomerulonephritis (44). HBV-MN podocytes and endothelial cells had increased expression of CTGF compared to normal controls, which suggested that CTGF may play a pathogenic role in renal fibrosis of HBV-MN.

Interestingly, the elevated expression of Leucine-rich alpha-2-glycoprotein 1 (*LRG1*) was displayed in HBV-MN endothelial cells (Supplementary Table 4). LRG1 has been identified as an inflammatory protein in human serum and highly expressed in various kinds of autoimmune diseases, including rheumatoid arthritis and lupus nephritis (45). Also, *NFKBIZ*, a gene highly expressed in endothelial cells in HBV-MN, which is a member of the IkappaB family of NF-kB regulators. It encodes NFkBIZ and is among the top up-regulated NF-kappa B-related genes at the mRNA level (Figure 2A). Moreover, we found that DEGs included overexpressed cellular proliferation-related genes such as *HSPA1A* and *IRF1* in kidney endothelial cells of HBV-MN (Supplementary Table 4). Interestingly, among these genes, Interferon regulatory factor 1 (IRF-1) was recently identified as a downstream target of TNF-mediated signal transduction in endothelial cells. As such, it might be highly related to the pathophysiology of HBV-MN through endothelial activation and dysfunction in the TNF signaling pathway (46).

To explore the pathogenesis between primary and secondary MN based on comparative genomics, intrinsic cell transcriptomes in the HBV-associated MN patient were compared to anti-PLA2R positive IMN transcriptomes, which also defined representative DEGs in the glomerulus (Figure 2D), tubules (Figure 2E), and immune cells (Figure 2F). Detailed analysis of DEGs in the glomeruli, tubules and immune cells are displayed in Supplementary Tables 7–9, respectively. Except for macrophages, T cells and monocytes could not be analyzed due to insufficient cell numbers. In addition, a deficiency of meaningful differential genes in plasma cells and mast cells might be caused by technical limitations in isolating insufficient corresponding cells since previous studies have confirmed their contributions in kidney diseases (47–49).

The level of *ESM1, ABI3BP*, and *HMGB1P5* was higher in HBV-MN than in IMN subjects in ECs (Figure 2D). We found that the genes *SPP1, TIMP3*, and *HLA-DRB5* were highly expressed in ECs in HBV-MN compared with IMN. These genes in endothelial cells were also upregulated when compared to HBV-MN with healthy subjects (Supplementary Tables 4, 7).

**Figure 3:**
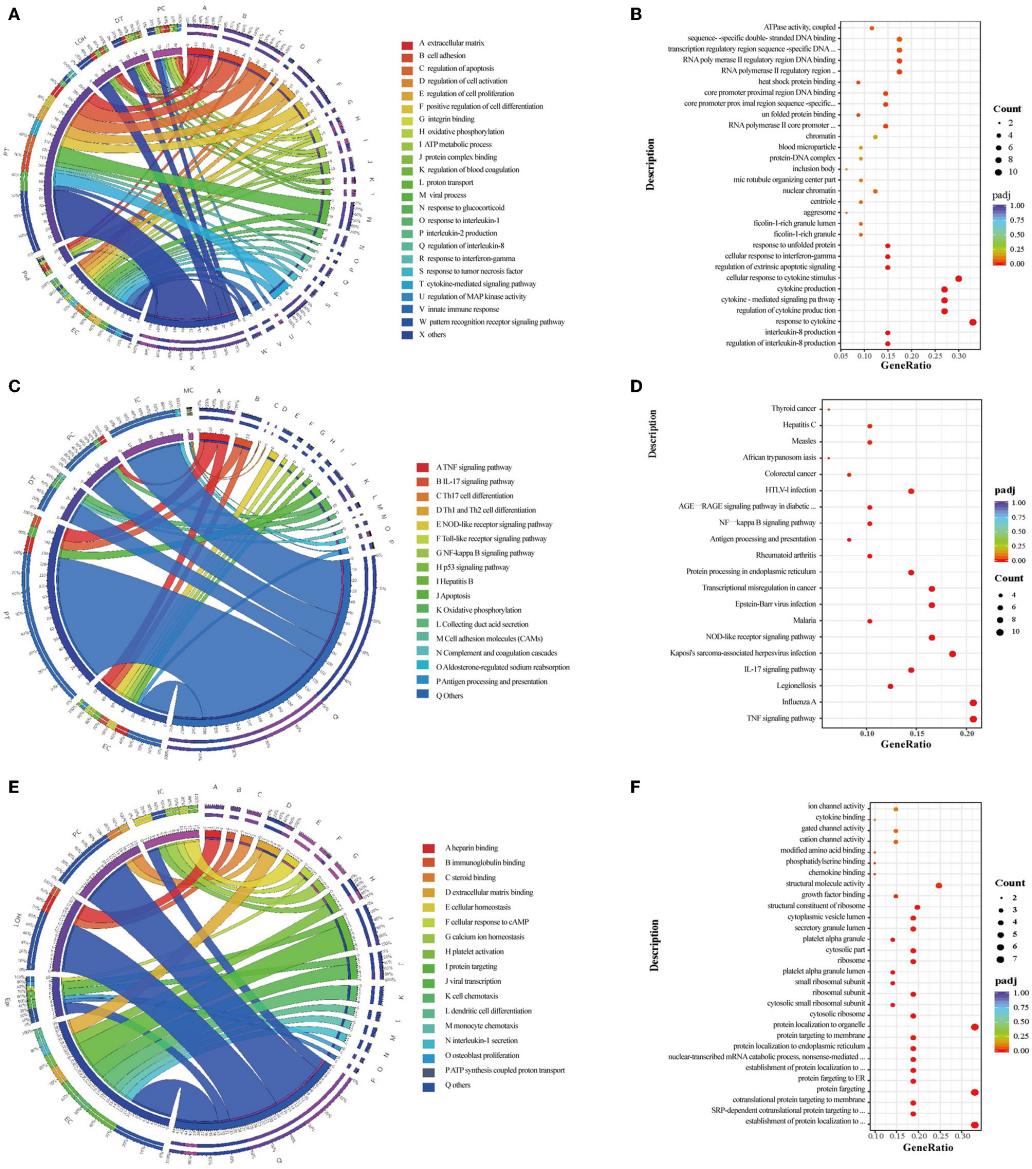
Enrichment analysis in the kidney cells between HBV-MN and healthy/IMN subjects. **(A,C)** GO and KEGG enrichment shows the biological processes or signal pathways involved in different kidney cells comparing the HBV-MN patients to the healthy donors. The left side of the circle represents different cell types, while the right side represents different biological processes or signaling pathways. The inner-circle represents gene numbers involved in cells or biological processes and signaling pathways, whereas the outer circle represents the proportion of each cell type in biological processes and signaling pathways or the proportion of biological processes and signaling pathways in kidney cells. **(B,D)** The bubble diagram shows the GO and KEGG enrichment analysis of endothelial cells comparing the HBV-MN patients to the healthy donors, respectively. **(E)** GO enrichment shows the biological processes involved in different kidney cells, comparing the HBV-MN patients to IMN subjects. **(F)** The bubble diagram shows the GO enrichment analysis of endothelial cells comparing the HBV-MN patients to IMN subjects.

GO enrichment analysis and GSVA revealed that the overexpressed DEGs between HBV-MN and healthy subjects were enriched in the extracellular matrix, cell adhesion, proliferation as well as a cytokine-mediated signaling pathway, whereas KEGG enrichment analysis showed oxidative phosphorylation, TNF signaling, IL-17 signaling, NF-kappa B signaling pathway, as well as antigen processing and presentation (Figures 3A,C, Supplementary Figure 4). In addition, extracellular matrix, viral gene expression, platelet degranulation and protein targeting to endoplasmic reticulum were enriched between HBV-MN and IMN by GO enrichment analysis (Figure 3E). Detailed information of enrichment analysis between HBV-MN and healthy subjects were listed in Supplementary Tables 10, 11.

Compared with healthy subjects, GO enrichment analysis in ECs revealed that the overexpressed DEGs in HBV-associated MN patient were enriched in viral process, innate immune response, leukocyte cell-cell adhesion, response to cytokine, and MAPK cascade (Figure 3B), whereas KEGG enrichment analysis demonstrated that the upregulated DEGs were involved in TNF signaling, IL-17 signaling, NOD-like receptor signaling, antigen processing and presentation, and NF-kappa B signaling pathway (Figure 3D). However, when compared with IMN patients, ECs in HBV-associated MN patients were more enriched in the process of platelet degranulation, viral gene expression, and protein targeting to endoplasmic reticulum, cell chemotaxis, as well as IL-1 and IL-6 secretion (Figure 3F). The increase in ribosomal protein synthesis may play a role in the pathogenesis of HBV-MN since HBV is well-known to enhance rRNA synthesis, leading to increased ribosome biogenesis and cell proliferation (50).

**Figure 4:**
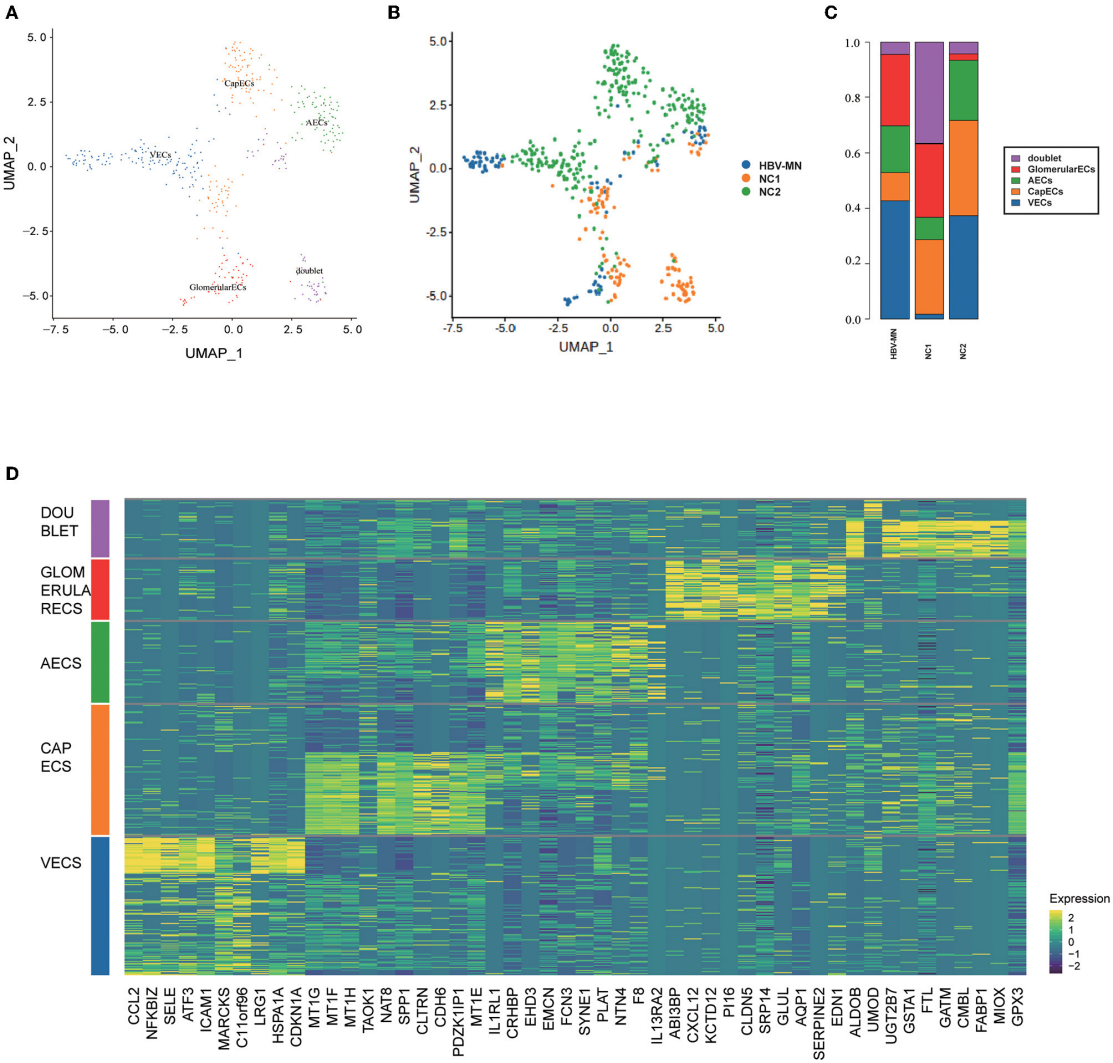
Distribution of EC subtypes, DEGs, and enrichment analysis. **(A)** Four distinct EC subtypes were visualized by UMAP plotting, and the color of the cells represents group origin. **(B)** UMAP plot of EC subtypes from different subjects of HBV-MN and healthy subjects. The color of cells reflected the individual origin. **(C)** Bar plots of the percent contribution of cell clusters in kidneys from different EC subjects. **(D)** Heatmap of the top 10 most DEGs in EC subtypes, which were then used to determine the cell lineage of each cluster.

To obtain more information on ECs, further division was performed and consequently identified four ECs subtypes, including glomerular ECs, vein ECs, artery ECs, and capillary ECs (Figures 4A–C), and the top 10 DEGs are displayed in Figure 4D. All subtypes of ECs were identified and shown in Supplementary Figure 2 with a feature plot of its marker genes. We further analyzed the DEGs in the healthy and HBV-MN subjects and found that the up-regulated DEGs in glomerular ECs were enriched in protein targeting to endoplasmic reticulum, viral transcription, viral gene expression, focal adhesion, regulation of fibroblast proliferation, and cadherin binding by GO enrichment (Supplementary Table 12).

### Tubular Cells From HBV-Associated MN Patients Were Enriched With Immune and Pro-inflammatory, Matrix Remodeling, and Related Signatures

A comparison of proximal tubule cells (PTs) between HBV-associated MN patients and healthy subjects exhibited upregulation of genes involved in IL-17 signaling, TNF signaling, cellular response to cytokine stimulus, and apoptosis. The PTs in HBV-MN had increased genes participating in TNF signaling (Figure 2E), IL-17 signaling pathway and apoptosis. We validated the genes *TNFAIP3*, and *JUN* in human tissues in kidney tubular cells (Supplementary Figure 3). Endothelial cells and proximal tubule cells highly expressed the A20 (TNFAIP3), an NF-kappaB-dependent stress response gene in ECs and SMCs with potent anti-inflammatory effects in both cell types through blockade of NF-kappaB pathway (51). *JUN* is a gene encoding a protein which is highly similar to the viral protein. In many cell types, c-Jun regulates the expression of genes involved in proliferation or inflammation; It has been suggested that c-Jun may play a role in regulating inflammation and/or fibrosis in human renal disease (52).

DEGs upregulated in distal tubule cells (DTs) such as *SDHD, CA2, CLCNKB, ATP6V0D2, NDFB3, and SLC4A1* were enriched in the oxidative phosphorylation and collecting duct acid secretion in GO and KEGG enrichment analysis.

The loop of Henle cells in HBV-MN had increased gene expression participating in the extracellular matrix. Moreover, genes participating in estrogen signaling pathway, including *GNAS, HSP90AB1, HSPA8, HSPA2*, and *FKBP5* were increased in the loop of Henle cells in HBV-MN. Also, genes participating in adherens junction, including *PLAU, HSPA1A, LMO7, CAPN2*, and *AHNAK* were increased in the loop of Henle cells in HBV-MN (Supplementary Tables 10, 11). Additionally, GO enrichment unveiled that upregulated genes, including *COL22A1, IGFBP7, EDN1, KNG1, and THBS1*, participated in the endoplasmic reticulum lumen in PCs. The KEGG analysis indicated that the key signaling pathway is oxidative phosphorylation with *MT-CO1, MT-ND4, MT-CO3, MT-CYB, MT-ND5*, and TNF signaling pathway (*JUNB, SOCS3, NFKBIA, ED N1, CXCL3*) in PCs.

Also, compared with IMN subjects, HBV-associated MN subjects showed an elevated expression of genes such as *JUN, FOS, JUND, DUSP1*, and *GADD45B* through the MAPK signaling pathway, osteoclast differentiation, estrogen signaling pathway, TNF signaling, and IL-17 signaling pathway in PTs (Supplementary Table 14). Further research is needed to clarify the role of MAPK signaling in HBV-associated-MN pathogenesis. Additionally, genes associated with collecting duct acid secretion and extracellular matrix binding in HBV-MN were also more abundant in principal cells (Supplementary Tables 13, 14). FOS was significantly overexpressed in proximal tubule cells of HBV-MN, which plays a crucial role in mesangial proliferation and glomerular sclerosis (53). GADD45B, a member of the growth arrest and DNA damage related gene family, may be involved in injury of podocytes in focal segmental glomerular sclerosis (FSGS) (54, 55).

### HBV-Associated MN Through Ligand-Receptor Interactions Analysis

**Figure 5:**
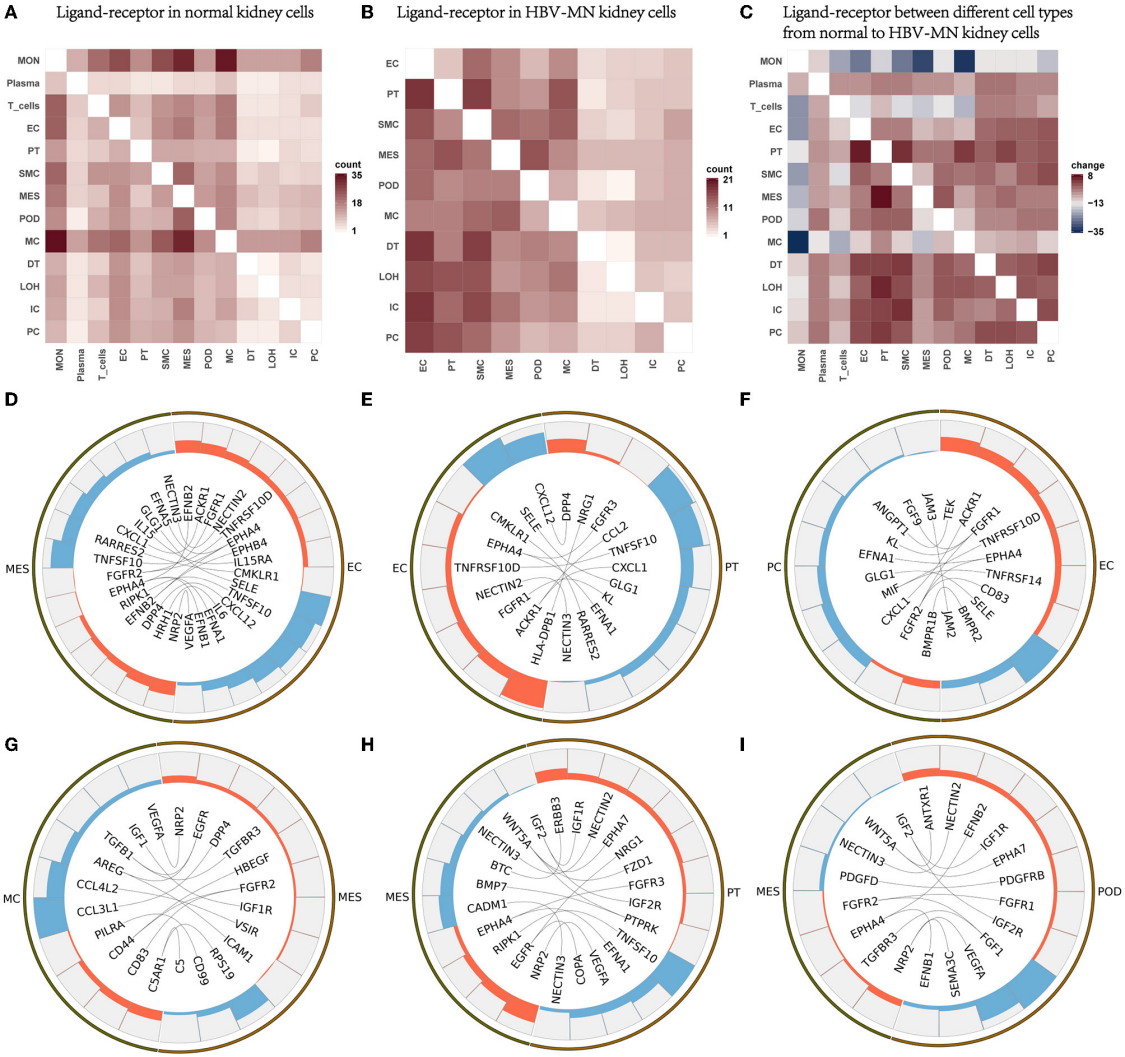
Ligand-receptor interactions in the kidney of HBV-MN and healthy subjects. **(A)** Ligand-receptor signaling pathways between cell clusters in healthy kidney cells. Cell-cell crosstalk frequency ranges from low (white) to high (red). **(B)** Ligand-receptor signaling pathways between cell clusters in HBV-MN kidney cells. Cell-cell crosstalk frequency ranges from low (white) to high (red). **(C)** Ligand-receptor between different cell types from normal to HBV-MN kidney cells. Cell-cell crosstalk frequency difference ranges from low (blue) to high (red). **(D–I)** Representative ligand-receptor interactions in EC-MES, EC-PC, EC-PT, MES-MC, MES-PT, MES-Pod. Lines represented interrelations between cells.

To explore the interactions and signaling network of different cell subsets in HBV-MN, we performed ligand-receptor analysis in healthy subjects, HBV-associated MN subjects, and changes from healthy subjects to HBV-associated MN subjects (Figures 5A–C). The results revealed that MESs, ECs, and PTs appear more active when interacting with other cells in HBV-associated MN subjects compared with healthy subjects. The potential interactions of receptors and ligands among different cell types of HBV-associated MN were examined (Figures 5D–I).

MESs, PCs, and PTs expressed *GLG1*, a self-glycosylating protein that primes glycogen synthesis (56), may interact with *SELE* from ECs, respectively (Figures 5D–F). We validated *SELE* at the protein level in kidney biopsy by immunofluorescence (Supplementary Figure 3). Mesangial cells and proximal cells expressing CXCL1 and CCL2, interact with ACKR1 in endothelial cells (Figures 5D,E), possibly promoting neutrophil/macrophage infiltration. CXCL1, a chemokine belonging to the class of CXC chemokines, causes damage to other cells in kidney diseases, in addition to its function as a neutrophil chemoattractant (57). Macrophages highly express TGF-β and become interaction pairs with *TGFBR3* in MESs (Figure 5G), possibly involved in cell recruitment and metastasis (58). More detailed information about the ligand-receptor interactions is shown in Figure 5.

## Discussion

In this study, kidney biopsy specimens from a patient with HBV-associated MN were deciphered using scRNA-seq, transcriptional maps were constructed. Then, we compared the DEGs as well as enrichment outcomes in healthy subjects or IMN subjects. Immunity and inflammatory pathway-related genes of ECs were enriched in glomerular cells with HBV-MN, including response to cytokine, antigen processing and presentation, apoptotic signaling pathway and response to interferon-gamma. Additionally, we found that the DEGs in most parenchymal cells were primarily enriched in the TNF signaling pathway, NOD-like receptor signaling pathway, IL-17 pathway, and its related-downstream NF-kappa B signaling pathway. Furthermore, TNF signaling, IL-17 signaling, NOD-like receptor signaling, and NF-kappa B signaling pathway were also confirmed to be enriched in DEGs in HBV-associated MN compared with anti-phospholipase A2 receptor (PLA2R)-positive IMN. Our study provided several novel insights into pathogenesis of human HBV-MN through detecting and analyzing cell subpopulations, cell-type-specific gene expression, and distinct signaling pathways.

We provided abundant information of cell-type-specific gene expression and distinct signaling pathways by analysis of DEGs as well as enrichment analysis. *ICAM-1, CTGF, IGFBP7*, and *HSPA5*, which were significantly expressed in all glomerular cells in HBV-MN compared with healthy donors in our study. Our results revealed that *ICAM-1* is highly expressed in ECs and PTs, possibly induced by various inflammatory cytokines in HBV-MN subjects. *ICAM-1*, a member of immunoglobulin receptor superfamily, plays a pivotal role in mediating intercellular interactions and inflammatory responses (58). It was reported that *ICAM-1* is highly expressed in endothelial cells of HBV-MN, IgA nephropathy and lupus nephritis (59, 60). *HSPA5*, an endoplasmic reticulum stress protein gene in podocytes, was upregulated by HBV infection, indicating the increase of endoplasmic reticulum stress in MN (41). Previous study has indicated that the upregulation of *HSPA5* expression participated in podocyte injury by immune complex formation (61). We speculated *HSPA5* might play an important role in the pathogenesis of HBV-MN, the possible mechanism includes endothelial-mesangial- podocyte-tubular crosstalk. A recent study showed that HSPA5 can also be involved in diabetic nephropathy by targeting RhoA and activating the PI3k/Akt pathway (62). PI3k/Akt pathway showed strong signaling in HBV-associated MN in our GSVA results. The aforementioned PI3K-Akt signaling pathway mediates the transformation of renal tubular epithelial cells and mesenchyme through a certain protein (63, 64). This mechanism may also act on HBV-associated MN, and the oxidative environment generated by it easily leads to a positive reaction with antiphospholipase A2, which provides a possible explanation for HBV-associated MN (65–67).

Upregulation of pro-inflammatory pathways were found in our study in glomerular cells, including TNF signaling pathway, IL-17 signaling pathway, Toll-like receptor signaling pathway, and NOD signaling pathways. Besides, multiple adaptive immune-related pathways were also elevated, for instance, antigen processing and presentation, apoptotic signaling pathway, response to interferon-gamma, and MAPK cascade. Among the numerous physiological and pathological functions played by the TNF signaling pathway are cell proliferation, apoptosis, differentiation, induction of inflammation, and regulatory processes of immune reactions, and it acts as a pathogenic signaling mechanism in other kidney diseases as well (68, 69). Besides, TNF signaling pathway activates multiple inflammatory downstream signaling and triggers the inflammatory cascades, including NF-kB and MAPK pathways (70, 71). It was reported that TNF allele G308A and the TNFd microsatellite poly-morphisms located downstream of the TNFA gene are strongly associated with the occurrence and initiation of MN (72, 73), TNFα can induce decohesion at the slit membrane with the disassembly of acting filaments, suggesting that TNFα might be involved in the induction or maintenance of glomerular barrier dysfunction (74). NF-kappa B plays a key role in regulating the immune response to infection. In particular, NF-kappa B signaling pathways were increased in ECs, indicating that the immune complex reaction occurs in the subendothelium in HBV-associated MN. It has been reported that the capillary wall lesion of HBV-associated glomerulopathy may be associated with the deposition of HBV-associated antigens, especially HBeAg and IgG1 antibodies (75). Hong et al. identified that MHBst167/HBx-induced NF-κB activation *via* the PKC/ERK pathway in hepatitis B virus-induced renal tubular cells undergoing apoptosis may be involved in virally induced pathogenesis (76). Here, we reported that HBx and MHBst might induce apoptosis ofrenal tubular epithelial cells by activating NF-kappa B. In addition, we also noticed extensive enrichment of NOD-like receptor signaling in glomerular and tubular cells, which was identified as critical intracellular pattern recognition receptor in innate immune responses and tissue homeostasis.

Endothelial cells, as the glomerular filtration barrier, have an intermediary role between the epithelial cells and white blood cells through interacting and responding to signals in the onset and progression of HBV-MN (64, 77, 78). The glomerular endothelium, which is highly fenestrated and covered by a rich glycocalyx, participates in the sieving properties of the glomerular filtration barrier and in the maintenance of podocyte structure. Glomerular endothelial homeostasis is crucial for maintaining glomerular structural integrity, anti-inflammatory and antithrombotic environments, and prevention of renal fibrosis (79). A recent study showed that all of their MN cases displayed glomerular endothelial injury (80). Furthermore, a prominent injury to the glomerular endothelium has been shown to be associated with a decline in eGFR and the formation of focal segmental glomerulosclerosis lesions in MN (80). Besides, Müller-Deile et al. reported the role of glomerular ECs—derived microRNAs targeting GBM proteins, may contribute to IMN by increasing GBM permeability, which would allow autoantibodies to reach the subpodocyte space (81).

In this study, we further characterized endothelial cells into four subtypes, and analyzed the molecular alterations and biological processes. As expected, glomerular ECs participate in viral transcription, viral gene expression, protein targeting to endoplasmic reticulum, focal adhesion, regulation of fibroblast proliferation, and cadherin binding. Previous studies reported that the activation and dysfunction of ECs, along with immune complexes mediated vascular injury and anti-phospholipid antibody-associated thrombotic events, were key determinants in vascular lesion development in LN (82). Chakraborty et al. found that modifying gene expression of endothelial cells could modulate leukocyte trans-endothelial migration and affect endothelial transcriptomes (83). HBV-induced glomerular ECs dysfunction is associated with the acquisition of proinflammatory and prothrombotic phenotypes that favor immune cell adhesion and infiltration, the destruction of fenestrated endothelial integrity, and increased endothelial-to mesenchymal transition. Activation of the endothelial inflammatory phenotype and the synthesis of proinflammatory factors are energy-dependent processes, and metabolic pathways in ECs can reprogram EC phenotypes independently (84).

A growing body of literature confirms that inflammation and fibrosis in the tubulo-interstitial space are common features of chronicity and disease development, and tubulointerstitial injury correlates more closely with renal prognosis than glomerular lesions. Tubular epithelial cells are also thought to play an important role in HBV-MN (63). According to our results, the genes overexpressed in proximal tubule cells are mainly associated with TNF signaling, IL-17 signaling, cellular response to cytokine stimulus, and apoptosis. IL-17 is mainly secreted by CD4^+^ T cells, which can induce epithelial cells, endothelial cells, and fibroblasts to synthesize and secrete IL-6, IL-8, G-CSF, and PGE2, and promote the expression of *ICAM-1* (85, 86). The cytokine IL-17 and its downstream signaling, also including NF-kappa B and MAPK signaling pathway, can increase the production of inflammatory cytokines and chemokines in kidney disease (87). Inflammatory cytokines are released, leukocytes are recruited, and kidney destruction is facilitated *via* IL-17 signaling. Of these, DEGs were significantly enriched in cytokine-cytokine receptor interaction. Hippo signaling pathway is a crucial pathway that regulates renal tubulointerstitial fibrosis, which shows positive in GSVA in HBV-MN (63, 77).

Comparison of LOH between HBV-associated MN patients and healthy subjects exhibited upregulation of genes involved in estrogen pathway. Studies have suggested that estrogen and estrogen receptors (ERs) play essential roles enriched in many physiological processes in the kidney (88–90). For instance, they facilitate the maintaining of mitochondrial homeostasis and modulation of the endothelin-1 (ET-1) system in the kidney. Estrogen participates in kidney repair and regeneration *via* its receptors and regulates phosphorus homeostasis *via* its receptors in the proximal tube-based receptor. Here we reported that altered or dysregulated estrogen/ERs nuclear signaling pathways might potentially contribute to HBV-MN.

Furthermore, in order to further validate the findings in our study, DEGs and enrichment analyses were compared between HBV-associated MN and anti-phospholipase A2 receptor (PLA2R)-positive IMN using scRNA-seq of renal cells. The results also showed that the genes overexpressed in the tubule cells of HBV-MN patients were mainly involved in inflammatory pathways such as TNF signaling, IL-17 signaling, and MAPK signaling pathway. It has been demonstrated that IL-17 and TNF pathways participate in the immune complex defense mechanism of both MN and HBV-associated-MN (91, 92). Endothelial cells highly expressed the A20 (TNFAIP3), an NF-kappaB-dependent stress response gene in ECs and SMCs with potent anti-inflammatory effects in both cell types through blockade of NF-kappaB pathway (51), which was validated on the protein level in the human tissues both in a HBV-MN patient and a IMN patient. Further research is needed to clarify the role of MAPK signaling in HBV-associated-MN pathogenesis.

The role of mesangial-podocytic-tubular cross-talk in the progression of various glomerulonephritis, including IgA nephropathy, has been well-recognized (93). To date, ligand-receptor (L-R) pairing analysis using single-cell or single-nucleus RNA sequencing (scRNAseq or snRNA-seq) gene expression datasets has been used to decipher cell-cell cross-talk within a local tissue or organ (94). Through intercellular receptor-ligand analysis, we found that *CCL3L1* was highly expressed in macrophages and interacted with *DPP4* in MESs. Previous studies reported that DPP4 inhibitors could ameliorate high-fat diet-mediated obesity-related glomerulopathy, which may relate to the improved insulin sensitivity and reduced local inflammation through inhibiting macrophage infiltration and IL-6 and TNF-α secretion (95). An importantly recent study indicateed that the differentiation of proximal tubule cells in kidney fibrosis and the interaction between these cells and basophils mediated by CXCL1, the chemokine responsible for basophil recruitment. A new therapeutic approach for the management of HBV-MN disease may be opened up through the contribution of basophils to renal fibrosis from proximal tubule cells (94, 96).

In addition, *SELE* was reported as an inflammatory marker, and played a role in kidney dysfunction with type 1 diabetes (97), which was overexpressed in the endothelial cells in HBV-associated MN patients. It was found that that immunosuppressive therapy contributed substantially to the improvement of endothelial dysfunction in adult-onset podocytopathy and primary membranous nephropathy (98). scRNA-seq was applied in this study and the analysis proved that *GLG1* expressed in mesangial cells, principal cells and proximal tubular cells, interacting with *SELE* in endothelial dysfunction (82, 83, 99). Hence, *GLG1* may participate in the pathogenic development of HBV-MN, providing a new insight into immune complexes-induced cellular crosstalk (100, 101).

There are some limitations in this study. First, the biopsy is from one HBV-MN patient, the results cannot be generalized, expanding the sample size would be helpful to reduce the impact of results caused by individual differences. Second, despite the role of immune and inflammatory genes of HBV-MN patients being identified (102), we obtained relatively limited immune cells, as such the information from other cells cannot be analyzed.

In conclusion, scRNA-seq is a feasible and effective method to analyze the gene information at the single-cell transcriptional level in HBV-MN patients. We constructed the transcription spectrum of HBV-MN for the first time as far as we know, and elucidated its biological activities in the extracellular matrix, viral process, and inflammation-related signal pathways, including innate and adaptive pathways. The ligand-receptor analysis emphasized the extensive communication of endothelial cells and mesangial cells or proximal tubule cells. These findings may provide new insights into the molecular mechanisms underlying HBV-associated MN tubulointerstitial injury and potential targets for the diagnosis and therapeutics of HBV-associated MN.

## Author's Note

Parts of the present study have been presented as a Mini-Oral at the 59th ERA-EDTA Congress, from May 19 to 22, 2022.

## Data Availability Statement

The original contributions presented in the study are publicly available. This data can be found here: Gene Expression Omnibus, GSE199850.

## Ethics Statement

The studies involving human participants were reviewed and approved by the Medical Ethics Committee of Xiangya Hospital, Central South University. The patients/participants provided their written informed consent to participate in this study.

## Author Contributions

YZ, XX, JO, and PE designed and supervised the project. XD, PJ, and TM offered assistance with permission and kidney tissue acquisition of IMN as well as healthy kidney donors. LY and WL detected and evaluated the renal histopathology, performed all biopsy dissociations, and single-cell experiments. YZ, CS, and LY conducted data analysis. LY, TM, WN, XL, XX, and YZ prepared the manuscript. JO and PE revised the manuscript. All authors contributed to the article and approved the submitted version.

## Funding

This work was funded by the National Key R&D Program of China (2020YFC2005000 to XX), the Key Research and Development Program of Hunan province (2020WK2008 to YZ), the Science and Technology Innovation Program of Hunan Province (2020RC5002 to JO), the Natural Science Foundation of Hunan Province (2021JJ31130 to YZ and 2020JJ6109 to CS), the Science and Technology Plan Project Program of Jiangxi Province (2021B238 to LY), the Science and Technology Plan Project of Jiujiang (S2021ZDYFN201 to LY), and Yiluqihang Shenmingyuanyang Medical Development and Scientific Research Fund Project on Kidney Diseases (SMYY20220301001 to YZ).

## Conflict of Interest

The authors declare that the research was conducted in the absence of any commercial or financial relationships that could be construed as a potential conflict of interest.

## Publisher's Note

All claims expressed in this article are solely those of the authors and do not necessarily represent those of their affiliated organizations, or those of the publisher, the editors and the reviewers. Any product that may be evaluated in this article, or claim that may be made by its manufacturer, is not guaranteed or endorsed by the publisher.
