# Supplementary figures and images for: Intrarenal Single-Cell Sequencing of Hepatitis B Virus Associated Membranous Nephropathy
Source: Front Med (Lausanne). 2022 Jul 22;9:869284. doi: 10.3389/fmed.2022.869284 (PMC9355751; doi:10.3389/fmed.2022.869284)

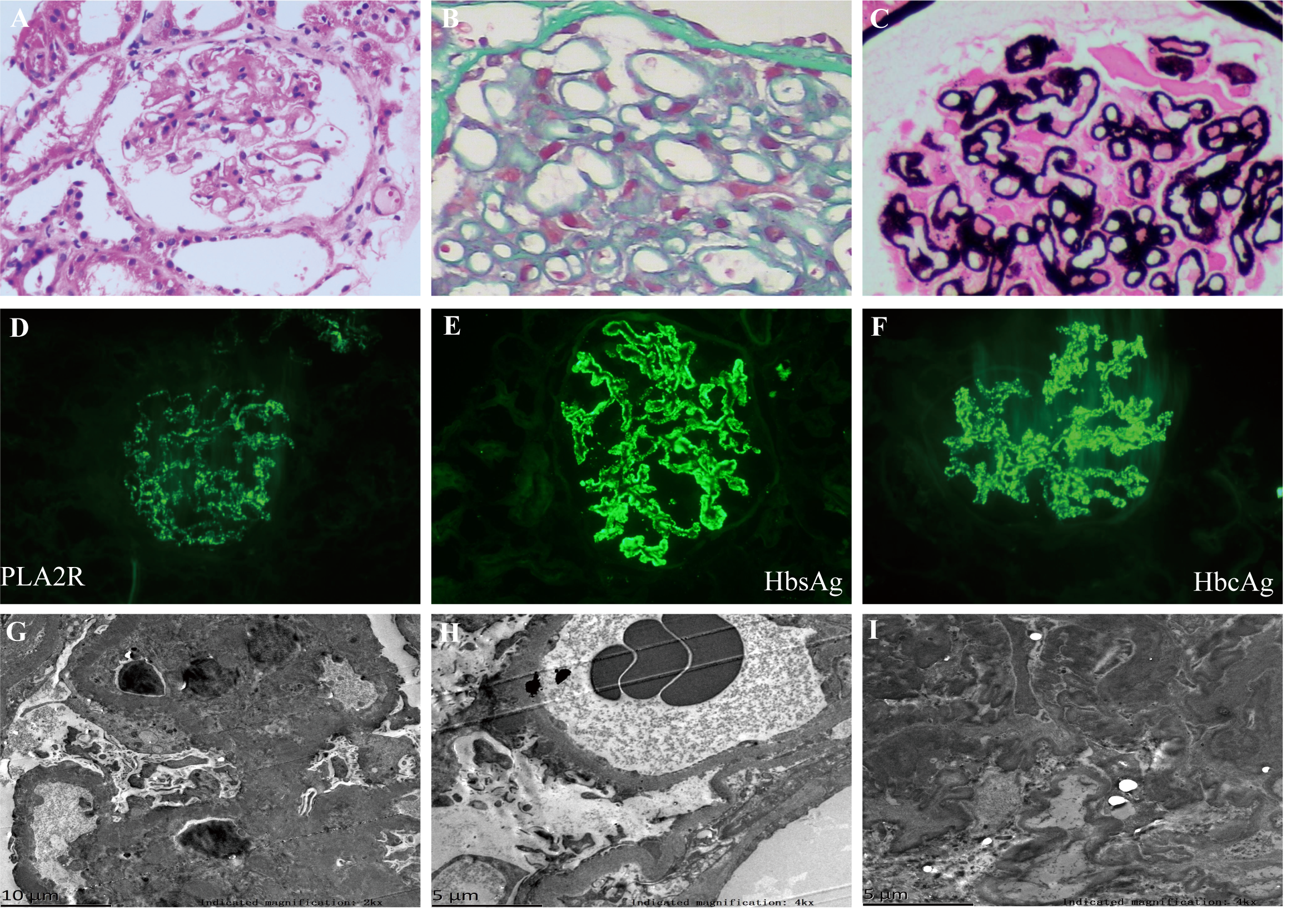

Supplement: Supplementary file 1 [file Data_Sheet_1.zip › 869284_SupMaterial (2)/Image 1.TIF]

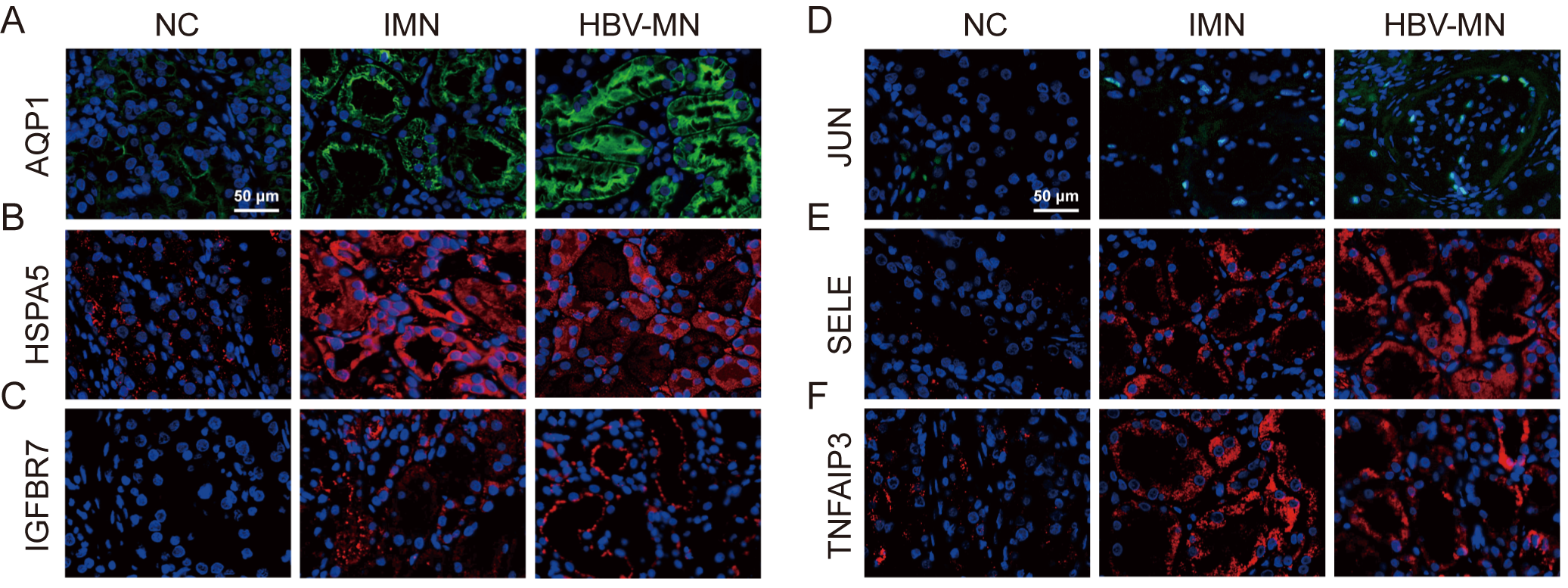

Supplement: Supplementary file 1 [file Data_Sheet_1.zip › 869284_SupMaterial (2)/Image 3.TIF]

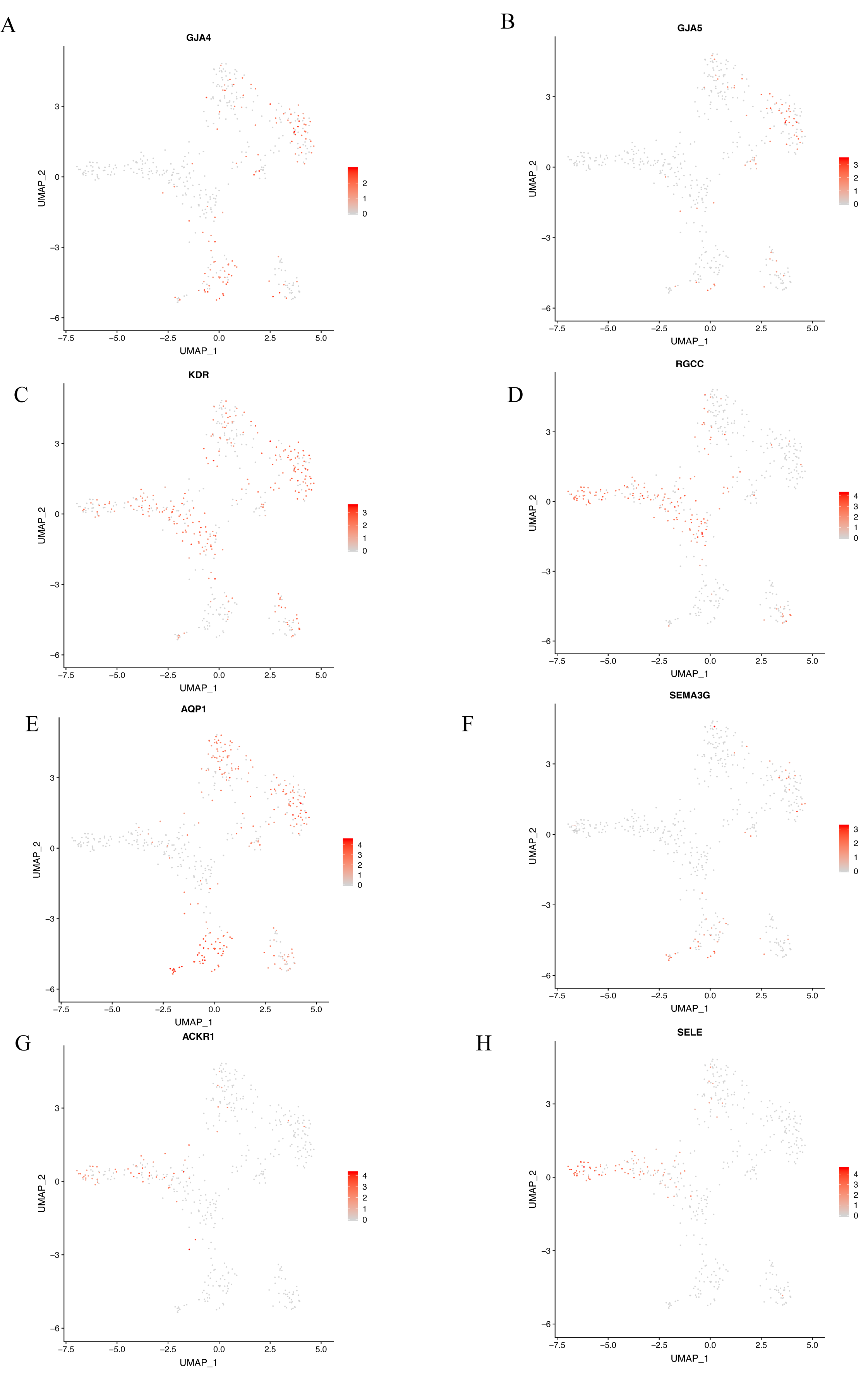

Supplement: Supplementary file 1 [file Data_Sheet_1.zip › 869284_SupMaterial (2)/Image 2.TIF]

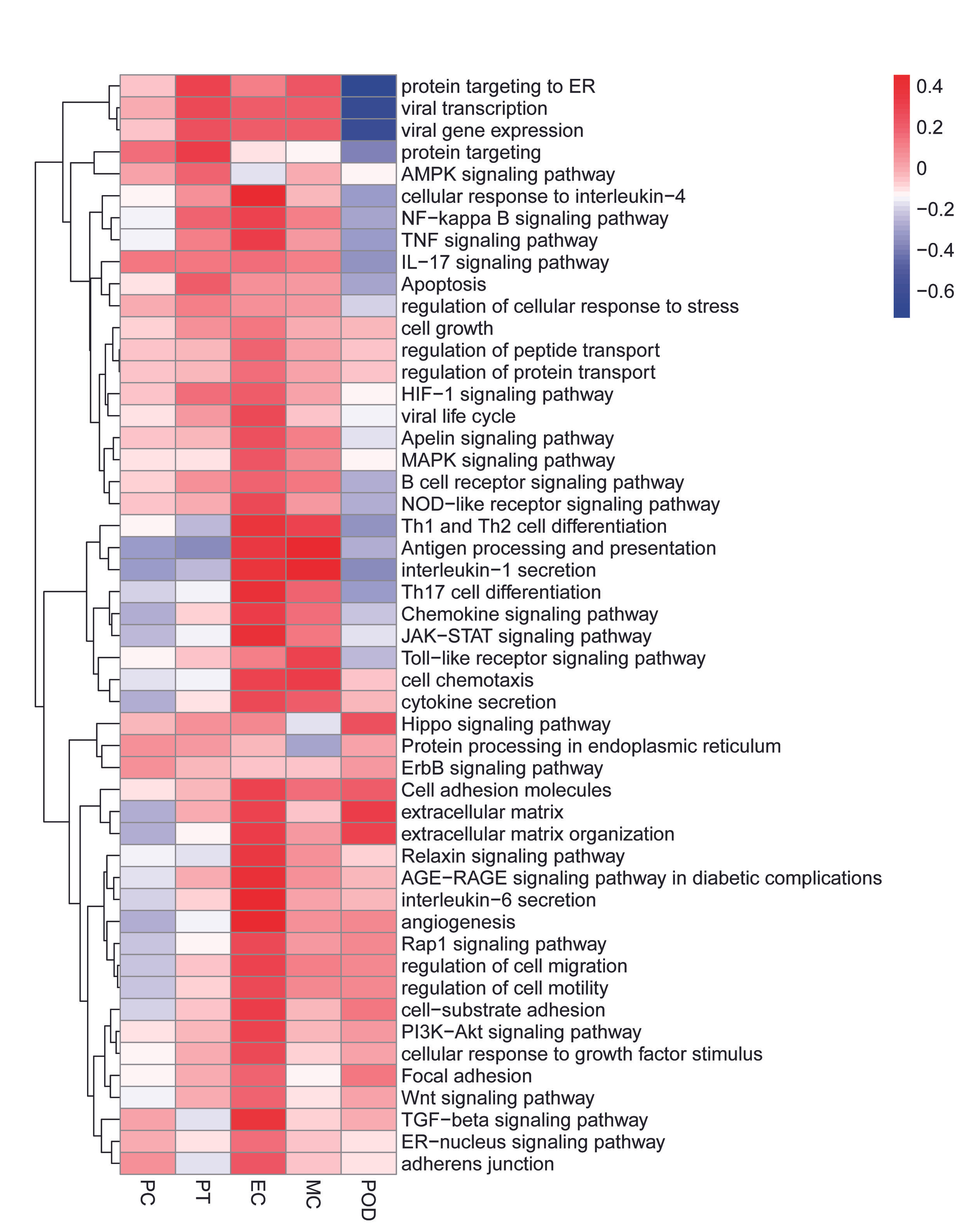

Supplement: Supplementary file 1 [file Data_Sheet_1.zip › 869284_SupMaterial (2)/Image 4.TIFF]
