# Supplementary material for: Intrarenal Single-Cell Sequencing of Hepatitis B Virus Associated Membranous Nephropathy
Source: Front Med (Lausanne). 2022 Jul 22;9:869284. doi: 10.3389/fmed.2022.869284 (PMC9355751; doi:10.3389/fmed.2022.869284)
Supplement: Supplementary file 1 [file Data_Sheet_1.zip › 869284_SupMaterial (2)/Table 15.DOC]

***Supplementary Materials***

**Supplementary Figure 1. Pathology characteristics of HBV-MN patients**

(A) Light microscopy shows heterogeneous thickening of the glomerular basement membrane accompanied by mesangial hypercellularity in HBV-MN patients (HE X200). (B)Masson staining shows there was a general accumulation of obvious deposition of fuchsinophilic substances under the epithelium (X400) (C)Periodic acid-silver methe-namine (PASM) staining shows a pattern of subepithelial spikes and the basement membrane and the formation of “spikes” with the silver stain. The internal vacuolizations in part of the GBM are apparent (X400). ((D) Granular staining for anti-PLA2R along the capillary wall by immunofluorescence (X200). (E) Positive HbsAg deposit along the capillary wall by immunofluorescence (X200).(F) Positive HbcAg deposit along the capillary wall by immunofluorescence (X200). (G, H and I) Electron microscopy shows that electron-dense substance was deposited under the glomerular epithelial cells and extensive podocyte fusion (G X5000 & H F X7000& I X10000).

**Supplementary Figure 2. Feature plot of the marker genes in EC subtype**

**Supplementary Figure 3. Immunofluorescence validated Protein in HBV-MN , IMN, and Health kidney biopsy**

**Supplementary Figure 4. The GSVA in different pathways in HBV-MN**

**Supplementary Table 1. Number of cells and viability for each sample**

**Supplementary Table 2. Cell number of distinct clusters in kidney from each subject**

**Supplementary Table 3. Top 20 marker genes of each cell type were shown in heatmap**

**Supplementary Table 4. DEGs in glomerulus cells from HBV-MN and control subjects**

Abbreviations were as follows: pct.1, the percentage of cells where the gene is detected in the HBV-MN patient; pct.2, the percentage of cells where the gene is detected in the control subjects; avg logFC, log fold-change of the average expression between the two groups, positive values indicate that the gene is more highly expressed in the first group.

**Supplementary Table 5. DEGs in tubular cells from HBV-MN and normal control subjects**

**Supplementary Table 6. DEGs in immune cells from HBV-MN and normal control subjects**

**Supplementary Table 7. DEGs in glomerulus cells from HBV-MN and IMN subjects**

**Supplementary Table 8. DEGs in tubular cells from HBV-MN and IMN subjects**

**Supplementary Table 9. DEGs in immune cells from HBV-MN and IMN subjects**

**Supplementary Table 10. GO enrichment of distinct clusters between HBV-MN and normal control subjects**

**Supplementary Table 11. KEGG enrichment of distinct clusters between HBV-MN and normal control subjects**

**Supplementary Table 12. GO and KEGG enrichment of distinct subtypes of ECs between HBV-MN and normal control subjects**

**Supplementary Table 13. GO enrichment of distinct clusters between HBV-MN and IMN**

**Supplementary Table 14. KEGG enrichment of distinct clusters between HBV-MN and IMN**

**Supplementary Table 1-14** were available at the supplementary materials as Excel.
